# Nivel de conocimiento sobre cáncer oral en los estudiantes de odontología de la Universidad Científica del Sur

**DOI:** 10.21142/2523-2754-0903-2021-072

**Published:** 2021-10-06

**Authors:** Paula Lucía Segura-Gaspar, Katherine Joselyn Atoche-Socola, Claudia Gabriela Otazú-Aldana

**Affiliations:** 1 Carrera de Estomatología, Universidad Científica del Sur. Lima, Perú. lucia9711@hotmail.com Universidad Científica del Sur Carrera de Estomatología Universidad Científica del Sur Lima Peru lucia9711@hotmail.com; 2 División de Rehabilitación Oral de la Universidad Científica del Sur. Lima, Perú. kattyas.22@gmail.com Universidad Científica del Sur División de Rehabilitación Oral Universidad Científica del Sur Lima Peru kattyas.22@gmail.com; 3 División de Odontopediatría de la Universidad Científica del Sur. Lima, Perú. cotazu@cientifica.edu.pe Universidad Científica del Sur División de Odontopediatría Universidad Científica del Sur Lima Peru cotazu@cientifica.edu.pe

**Keywords:** cáncer oral, nivel de conocimiento, odontología, Oral cancer, level of knowledge, dentistry

## Abstract

**Objetivo:**

El objetivo de este estudio fue determinar el nivel de conocimiento sobre cáncer oral de estudiantes de tercero, cuarto y quinto año de la carrera profesional de Odontología en la Universidad Científica del Sur, en el año 2020.

**Materiales y métodos:**

Se realizó un cuestionario sobre conocimientos de cáncer oral a 166 alumnos que cursaban tercero, cuarto y quinto año de la carrera de Odontología. Este cuestionario consistió en 24 preguntas de opción múltiple acerca de epidemiología, etiopatogenia, diagnóstico, tratamiento y complicaciones sobre cáncer oral.

**Resultados:**

Se encontró una asociación significativa entre el nivel de conocimiento sobre etiopatogenia del cáncer oral y el año de estudio.

**Conclusión:**

Los alumnos de tercer año tuvieron el mayor porcentaje en relación con nivel de conocimiento sobre etiopatogenia.

## Abstract (en)

**Objective:**

The objective of this study was to determine the level of knowledge about oral cancer of third, fourth- and fifth-year students of dentistry at the Cientifica del Sur University in 2020.

**Materials and methods:**

A questionnaire on knowledge of oral cancer was carried out in 166 students in the third, fourth and fifth year of dentistry. This questionnaire consisted of 24 multiple-choice questions about epidemiology, etiopathogenesis, diagnosis, treatment, and complications.

**Results:**

A significant association was found between the level of knowledge about the etiopathogenesis of oral cancer and the year of study.

**Conclusion:**

Third-year students had the highest percentage in relation to the level of knowledge about etiopathogenesis.

## INTRODUCCIÓN

El cáncer oral se encuentra entre los tumores malignos más comunes en personas de todo el mundo, principalmente con un mal pronóstico ^1^^,^^2^. Este tipo de neoplasia maligna surge en el labio o la cavidad oral. Se define como carcinoma de células escamosas, ya que, en el área dental, el 90% de los cánceres tienen un origen histológico en las células escamosas ^3^^,^^4^. Gran parte de los tumores malignos de la cavidad oral, la orofaringe y la faringe son originados a partir de lesiones premalignas como la leucoplasia y la eritroplasia ^5^. El carcinoma epidermoide es la neoplasia con mayor prevalencia de la cavidad oral. El carcinoma de células escamosas representa el 5% de todos los cánceres en los hombres y el 2% en las mujeres ^6^.

En Latinoamérica, el cáncer ocupa el tercer lugar entre las causas de muerte. En el Perú, el Registro de Cáncer de Lima evidenció que las tasas de incidencia para todos los cánceres en ambos sexos, desde 2009 hasta el 2018, registró 2869 nuevos casos de cáncer oral ^7^. El Instituto Nacional de Estadística e Informática (INEI) reportó el 2015 que, de cada 10 personas cuya edad bordea de 15 años a más, dos afirmaron fumar al menos un cigarrillo; práctica que es más frecuente en los hombres, en comparación con las mujeres. Como consecuencia, se ha reportado que en el Perú, en el 2015, el cáncer oral y de faringe conforman el 1,2% de la mortalidad causada por el tabaquismo ^8^. En un trabajo de investigación realizado en la Universidad Mayor de San Marcos se concluyó que los estudiantes que habían observado algún caso de cáncer oral tenían mayor conocimiento que aquellos que no lo habían hecho ^6^.

Un entrenamiento y una conciencia adecuados, como base, son importantes para reducir la mortalidad causada por el cáncer oral ^9^. Para alcanzar esto, es importante que los dentistas tengan suficiente conocimiento y conciencia sobre la detección y el diagnóstico precoz, por lo que el plan de estudios debe estar dirigido a la comunidad universitaria de manera que los estudiantes puedan conocer el estado real y las necesidades de las personas ^10^^,^^11^.

Entre los estudiantes de odontología de la Universidad de Kuwait, se evaluó el conocimiento sobre factores de riesgo, conceptos de diagnóstico y aspectos de atención clínica de casos de cáncer oral. Se encontró que cerca del 68,5% de los estudiantes referirían a los pacientes con sospecha de lesiones malignas solo después de 6 meses, durante los cuales la lesión seguiría desarrollándose ^12^. Esto resulta sorprendente dada la importancia de derivar al paciente tempranamente ante la sospecha de lesiones premalignas. En un estudio realizado en estudiantes de odontología yemeníes, se demostró la necesidad urgente de un programa estructurado de enseñanza, con un mayor enfoque en el diagnóstico de signos y síntomas de cáncer oral y lesiones precancerosas ^13^.

A lo largo de sus estudios, los alumnos la carrera de Estomatología de la Universidad Científica del Sur abordan el tema de cáncer oral en diferentes cursos. En segundo año, llevan los cursos de patología general y oral, anestesiología y cirugía, en tercer año, el curso de medicina estomatológica; en cuarto año, el curso de cirugía y traumatología I; y en quinto año, prótesis somática, cirugía y traumatología II, así como internado estomatológico.

El objetivo de este trabajo fue determinar cuál es el nivel de conocimiento sobre cáncer oral en estudiantes de tercero, cuarto y quinto año de la carrera profesional de Estomatología de la Universidad Científica del Sur. 

## MATERIALES Y MÉTODOS

El estudio fue observacional de tipo descriptivo transversal, ya que se encuestó a alumnos que se encontraban cursando tercero, cuarto y quinto año de odontología, entre los meses de octubre y noviembre del 2020, en la Universidad Científica del Sur.

La muestra final estuvo conformada por 166 alumnos. Entre los criterios de inclusión se consideró a los alumnos que formaron parte del estudio y que fueran del tercero, cuarto y quinto año. Como criterios de exclusión estuvieron la negativa de los alumnos a formar parte del estudio y los casos en los que se encontraban cursando otros ciclos distintos a los mencionados en los criterios de inclusión.

Para la recolección de datos, se utilizó un cuestionario estructurado, el cual fue elaborado por la UNMSM y validado por un grupo de expertos. Se utilizó el análisis de fiabilidad, el cual dio como resultado un alfa de Cronbach de 0,762 ^6^. Este cuestionario consistió en 24 preguntas de opción múltiple acerca de epidemiología, etiopatogenia, diagnóstico, tratamiento, complicaciones y prevención sobre cáncer oral. El cuestionario se realizó en la aplicación Google Forms. Se ingresó a las clases virtuales con la autorización del docente encargado y se les explicó a los alumnos en qué consistía el estudio. Dentro del cuestionario se adjuntó el consentimiento informado para que el alumno aceptara o no formar parte del estudio.

La variable de estudio principal fue el nivel de conocimiento sobre cáncer oral, la cual se enfoca en cinco dimensiones, cada una de las cuales tuvo preguntas asignadas con una alternativa correcta a la cual se le asignó un puntaje.

La sumatoria de los puntajes de cada dimensión representó el nivel de conocimiento del alumno sobre cáncer oral. Un puntaje menor o igual a 12 puntos equivale a un nivel bajo; de 13 a 15 puntos, a un nivel regular; e igual o mayor a 16 puntos, a un nivel alto.

Los datos fueron vaciados en una hoja de Microsoft Excel (2016) para ser codificados y, posteriormente, trasladados a la base de datos del programa estadístico SPSS. El análisis bivariado se realizó mediante la prueba de chi cuadrado o exacta de Fisher. Todo se analizó con un nivel de significancia p < 0,05.

## RESULTADOS

De acuerdo con el nivel de conocimiento sobre cáncer oral según el año de estudio, se observó que tercer año tuvo el nivel de conocimiento más alto en comparación con cuarto y quinto año. No se presentó una asociación estadísticamente significativa, con un el valor de p = 0,4 (Tabla 1).

**Tabla 1:** Nivel de conocimiento sobre cáncer oral, según el año de estudio

Nivel de conocimiento sobre cáncer oral	Tercer año		Cuarto año		Quinto año		Total		p
	N.o	%	N.o	%	N.o	%	N.o	%	
Bajo	4	36,4%	4	36,4%	3	27,3%	11	100%	0,4
Regular	21	56,8%	9	24,3%	7	18,9%	37	100%	
Alto	47	39,8%	36	30,5%	35	29,7%	118	100%	
Total	72	43,4%	49	29,5%	45	27,1%	166	100%	

Por otro lado, al evaluar el nivel de conocimiento sobre epidemiología del cáncer, según el año de estudio, se evidenció que no hay un nivel alto de conocimiento. Además, el tercer año presentó el porcentaje más bajo de conocimiento (45%). Se observó que no existe una asociación estadísticamente significativa, con un valor de p = 0,4 (Tabla 2).

**Tabla 2:** Nivel de conocimiento sobre epidemiología del cáncer, según el año de estudio

Nivel de conocimiento sobre epidemiología del cáncer oral	Tercer año		Cuarto año		Quinto año		Total		p
	N.o	%	N.o	%	N.o	%	N.o	%	
Bajo	60	45,5%	36	27,3%	36	27,3%	132	100%	0,4
Regular	12	35,3%	13	38,2%	9	26,5%	34	100%	
Alto									
Total	72	43,4%	49	29,5%	45	27,1%	166	100%	

Asimismo, en cuanto al nivel de conocimiento sobre etiopatogenia del cáncer oral, según el año de estudio, se evidenció que el tercer año tuvo un mayor porcentaje en el nivel regular de conocimiento. Además, presentó el mayor porcentaje (40%) de nivel de conocimiento alto sobre etiopatogenia. Sin embargo, se evidenció que quinto año tuvo el 40,4% de nivel regular de conocimiento y el 21,5% tuvo un nivel de conocimiento alto. Se encontró asociación estadísticamente significativa, con un valor de p = 0,005 (Tabla 3).

**Tabla 3:** Nivel de conocimiento sobre etiopatogenia del cáncer oral, según el año de estudio

Nivel de conocimiento sobre etiopatogenia del cáncer oral	Tercer año		Cuarto año		Quinto año		Total		p
	N.o	%	N.o	%	N.o	%	N.o	%	
Bajo	10	38,5%	10	38,5%	6	23,1%	26	100%	0,005
Regular	24	51,1%	4	8,5%	19	40,4%	47	100%	
Alto	38	40,9%	35	37,6%	20	21,5%	93	100%	
Total	72	43,4%	49	29,5%	45	27,1%	166	100%	

Con respecto al nivel de conocimiento sobre diagnóstico del cáncer oral, según el año de estudio, se evidenció que el tercer año tuvo el mayor porcentaje (42,2%) en nivel de conocimiento alto. No existió una asociación estadísticamente significativa, con un valor de p = 0,65 (Tabla 4).

**Tabla 4:** Nivel de conocimiento sobre diagnóstico del cáncer oral, según el año de estudio

Nivel de conocimiento sobre diagnóstico del cáncer oral	Tercer año		Cuarto año		Quinto año		Total		p
	N.o	%	N.o	%	N.o	%	N.o	%	
Bajo	3	75%	0	0%	1	25%	4	100%	0,65
Regular	15	44,1%	11	32,4%	8	23,5%	34	100%	
Alto	54	42,2%	38	29,7%	36	28,1%	128	100%	
Total	72	43,4%	49	29,5%	45	27,1%	166	100%	

En el caso del nivel de conocimiento sobre tratamiento y complicaciones del cáncer oral, según el año de estudio, se observó que tercer año tuvo el 43,8% con nivel de conocimiento alto y el 50,8% con un nivel bajo. No existió una asociación estadísticamente significativa, con un valor de p = 0,26 (Tabla 5).

**Tabla 5:** Nivel de conocimiento sobre tratamiento y complicaciones del cáncer oral, según el año de estudio

Nivel de conocimiento sobre tratamiento y complicaciones del cáncer oral	Tercer año		Cuarto año		Quinto año		Total		p
	N.o	%	N.o	%	N.o	%	N.o	%	
Bajo	33	50,8%	20	30,8%	12	18,5%	65	100%	0,26
Regular	32	37,6%	26	30,6%	27	31,8%	85	100%	
Alto	7	43,8%	3	18,8%	6	37,5%	16	100%	
Total	72	43,4%	49	29,5%	45	27,1%	166	100%	

## DISCUSIÓN

El conocimiento sobre etiología, etiopatogenia y factores de riesgo sobre cáncer oral es de gran importancia tanto para la población como para los clínicos, a fin de planificar medidas preventivas y diagnosticarlas a tiempo ^14^. El rol del odontólogo es de vital importancia en la prevención, el diagnóstico y el tratamiento del cáncer oral. En lo que se refiere a la prevención primaria, el odontólogo debe aconsejar al paciente que lleve una vida saludable, lo que significa evitar el consumo de tabaco y alcohol, e incentivar una alimentación balanceada ^15^.

El instrumento para determinar el nivel de conocimiento sobre cáncer oral fue un cuestionario validado por un juicio de expertos, realizado por Izaguirre en la Universidad Nacional Mayor de San Marcos ^6^. El cuestionario fue de opción múltiple y constó de 24 preguntas sobre epidemiología, etiopatogenia, diagnóstico, tratamiento, complicaciones y prevención del cáncer oral. 

Se han realizado diversos estudios que evalúan el nivel de conocimiento sobre cáncer oral en alumnos de odontología, en los cuales se han utilizado como instrumentos los cuestionarios. Entre los revisados, la mayoría de los cuestionarios realizan preguntas sobre el tipo de cáncer oral más prevalente, la edad y el género, los factores de riesgo, los signos y síntomas que presentan los pacientes con cáncer oral, lesiones premalignas y su localización más común. Esto es similar al estudio realizado por Carter, en la Universidad de Dundee, a estudiantes de medicina y odontología, y en el que, además de las preguntas mencionadas, se le formuló otras para que den su opinión sobre la suficiencia de sus conocimientos en detección y prevención del cáncer oral, su intención de adquirir más información y de qué forma preferirían obtener esa información ^16^.

En nuestro estudio, se evaluó el nivel de conocimiento sobre cáncer oral en los alumnos de tercero, cuarto y quinto año, similar al estudio de Keser en Turquía, pero este solo incluyó a los alumnos de tercer y quinto año ^17^. Asimismo, la investigación de Costamagma en Tacna evaluó a los alumnos de cuarto y quinto año de estudio, al igual que el estudio realizado por Cruz en Piura, en el cual se evaluó a los alumnos de cuarto y quinto año (7.o a 10.o ciclo) ^18^^,^^19^. Radman, en Croacia, incluyó en su estudio a los alumnos de cuarto y quinto año ^1^. García, en Cuba, también realizó su investigación en alumnos de tercero, cuarto y quinto año de la facultad de Estomatología ^20^. El motivo por el cual se decidió incluir en nuestro estudio a los alumnos de tercer año fue porque los estudiantes de la facultad de Estomatología de la Universidad Científica del Sur empiezan a recibir cursos relacionados con patología oral desde tercer ciclo de la carrera, a diferencia de los alumnos de las universidades incluidas los estudios antes mencionados.

En el 2014, un estudio realizado por Costamagna evaluó de una muestra de 159 estudiantes de odontología de tres universidades de Tacna, quienes se encontraban cursando cuarto y quinto año, y mostraron un nivel regular de conocimiento sobre cáncer oral ^18^. Asimismo, en el 2017, un estudio realizado en la Universidad César Vallejo, en Piura, con una muestra de 102 estudiantes de cuarto y quinto año de la carrera de Odontología, halló que el nivel de conocimiento fue regular ^19^.

En nuestro estudio, los alumnos de cuarto y quinto año presentaron un nivel de conocimiento bajo y alto, respectivamente, sobre cáncer oral. En el estudio realizado por García, en Cuba, en el 2019, a los alumnos de la carrera de Estomatología, los estudiantes de quinto año obtuvieron el mayor porcentaje (8,8%) en nivel alto sobre conocimiento de cáncer oral, lo cual difiere de nuestro estudio, en el cual fueron los alumnos de tercer año quienes tuvieron el porcentaje más alto (39,8%) ^20^. Esto también difiere con nuestra hipótesis, en la cual se esperaba que los alumnos de quinto año tuvieran un nivel de conocimiento alto sobre cáncer oral. Esto podría deberse a que los alumnos de quinto año se encuentran más enfocados en los cursos de operatoria dental, mientras que los de tercer año acaban de recibir los cursos de patología oral y general, por lo que tienen los conocimientos más presentes. Por otro lado, en el estudio de Fotedar, en India, los alumnos de tercero, cuarto y quinto año se encontraron en un nivel alto de conocimiento, siendo tercer año el que tuvo un mayor porcentaje (80,5%) ^21^. Se diferencia del estudio de Porras, en el cual los alumnos de cuarto año mostraron un nivel bajo y los de quinto año, un nivel regular de conocimiento sobre cáncer oral ^22^.

En la sección de epidemiología, los alumnos fueron interrogados sobre la edad, el género en el cual se presentan con más frecuencia los casos de cáncer oral y el tipo más prevalente. En nuestros resultados, encontramos que los alumnos de cuarto año tienen un nivel de conocimiento regular, lo cual difiere de los resultados de la investigación de Costamagna, en la cual los alumnos presentaron un nivel de conocimiento bajo ^18^. Asimismo, los alumnos de quinto año presentaron un nivel bajo de conocimiento, lo cual coincide con la investigación de Costamagna, en la cual también los alumnos de quinto año presentaron un nivel bajo en la dimensión de epidemiología ^18^. Esto coincide con el estudio de Izaguirre, en el cual los alumnos de quinto año se encontraron dentro de un nivel regular y bajo de conocimiento ^6^. 

Por otro lado, en el estudio de Porras, los alumnos de cuarto y quinto año mostraron un nivel bajo de conocimiento ^22^. Además, en nuestra investigación no se observó un nivel alto en conocimiento en ninguno de los años de estudio, lo que podría deberse a que, por la pandemia, las clases son llevadas de manera virtual. En este sentido, los casos clínicos que evalúan los alumnos sobre cáncer oral se están presentando mediante imágenes o videos. Previo a la pandemia, en las horas prácticas clínicas de ciertos cursos, se solía acudir a centros hospitalarios. Durante las rotaciones en los centros hospitalarios, los alumnos podían observar a pacientes con patologías orales, con lo que reforzaban su aprendizaje.

Al evaluar el nivel de conocimiento en cáncer oral, en la sección de etiopatogenia, se evaluó los factores de riesgo de cáncer oral. Este estudio se realizó en alumnos de odontología, ya que es importante que al ser personal médico y futuros dentistas identifiquen las lesiones epiteliales orales potencialmente premalignas para evitar el retraso en el diagnóstico ^23^. Se encontró una asociación significativa entre el año de estudio y el nivel de conocimiento sobre etiopatogenia, con un valor de p = 0,005. Esto difiere del estudio de Rahman en estudiantes de odontología de los Emiratos Árabes Unidos, en el que no se encontró la esperada asociación estadísticamente significativa al evaluar el nivel de conocimiento sobre factores de riesgo de cáncer oral según el año de estudio ^24^.

En los resultados de nuestra investigación, el nivel de conocimiento sobre etiopatogenia fue regular ^(^51,1%) en tercer año, bajo (38,5%) en cuarto año y regular (40,4%) en quinto año. Esto coincide con el estudio de Izaguirre, en el que los alumnos de quinto año tuvieron un nivel regular (65%) de conocimiento ^6^, pero difiere del estudio de Costamagna, en el cual el mayor porcentaje (47,8%) de alumnos presentó un nivel alto ^18^. En el estudio de Cannick, en Carolina del Sur, los alumnos de tercer año obtuvieron un nivel bajo (50%); los de cuarto año, un nivel alto (54.5%); y los de quinto año, un nivel entre regular y alto (45%), siendo cuarto año el de mayor porcentaje (54,5%) en el nivel alto. En nuestro estudio, en cambio, los alumnos de tercer año obtuvieron el mayor porcentaje (42,2%) en el nivel alto.^25^ Por otro lado, en el estudio de Boroumand, en Maryland, los alumnos de cuarto año fueron los que obtuvieron el mayor porcentaje (80,8%) en el nivel alto ^26^.

En la dimensión que corresponde a diagnóstico, se evaluó el conocimiento sobre signos y síntomas, lesiones de riesgo y la localización más frecuente del cáncer oral. El nivel de conocimiento sobre diagnóstico fue bajo en tercer año (75%), regular en cuarto año (32,4%) y alto en quinto año (28,1%), tal como en el estudio de Costamagna en Tacna, en el cual el 36,5% de los alumnos presentaron un nivel alto y el 35,8% se encontraban en un nivel de regular ^18^. Asimismo, en el estudio de Cannick, los alumnos de tercer año también obtuvieron un nivel bajo (60,4%), alto en cuarto año (65,9%) y alto en quinto año (75%) ^25^. Esto se diferencia del estudio de Izaguirre en Lima, en el que se encontraron los mejores puntajes en la sección de diagnóstico, pues el 47 % de los alumnos tuvo un nivel de conocimiento regular y el 41%, un nivel de conocimiento alto ^6^. Lo mismo ocurre en el estudio de Porras, en el cual los alumnos de cuarto año tuvieron un nivel regular (61,1%), al igual que los de quinto año (69,2%) ^22^. 

Por otro lado, Bobby, en la Universidad de Kuwait, encontró que los alumnos de tercero, cuarto y quinto año obtuvieron un nivel alto en diagnóstico, siendo quinto año los del mayor porcentaje (87%) en nivel alto ^12^. En el estudio de Boroumand en Maryland, los alumnos de tercer año tuvieron un nivel alto (48,4%); los de cuarto año, un nivel regular (53,4%), y los de quinto año, un nivel bajo (37,5%), siendo tercer año los que presentaron el mayor porcentaje (48,4%) en el nivel alto ^26^.

El nivel de conocimiento en estudiantes de odontología sobre el tratamiento y complicaciones del cáncer oral fue bajo en tercero, bajo en cuarto año y alto en quinto año, al igual que en el estudio de Costamagna, el cual halló que los alumnos de cuarto y quinto año también presentaron un nivel bajo en esta sección ^18^. Lo mismo sucede en el estudio de Porras, en el cual los alumnos de cuarto año tuvieron un nivel bajo y los de quinto año, un nivel regular ^22^. Es importante solicitar información sobre antecedentes de los pacientes que presenten lesiones sospechosas para obtener un buen diagnóstico y el tratamiento oportuno, en caso de que la curación de una lesión no se produzca en una semana ^1^.

Una limitación del estudio fue la muestra, ya que no fue homogénea para tercero, cuarto y quinto año, ya que los alumnos de tercer año fueron quienes tuvieron un número representativo. Además, podemos observar en las diferentes tablas que los alumnos de tercer año mostraron los mayores porcentajes en el nivel alto en las diferentes dimensiones evaluadas, lo que podría deberse a que las horas lectivas en los cursos sobre patología oral son mayores en los primeros ciclos de la carrera. Esto constituye una diferencia con otras universidades, en las cuales se llevan cursos como medicina estomatológica desde séptimo hasta décimo ciclo, de manera que se refuerzan los conocimientos sobre cáncer oral. Esto coincide con los resultados del estudio realizado por Izaguirre, en la cual los alumnos de último año de la Universidad Nacional Mayor De San Marcos, obtuvieron el mayor porcentaje de nivel de conocimiento sobre cáncer oral ^6^. Por tal motivo, sería ideal que, en los diferentes cursos, las horas lectivas en las que se toquen temas de cáncer oral a lo largo de la carrera sean mayores. 

Se sugiere que este estudio se extienda también a los alumnos de posgrado de la universidad, para conocer su nivel de conocimiento sobre cáncer oral y, de ser necesario, reforzarlo mediante capacitaciones, ya que es importante que sigan actualizando sus conocimientos para instruir a los pacientes a que abandonen los hábitos de riesgo que contribuyen a la aparición de cáncer oral. 

## CONCLUSIÓN

En el estudio, se encontró diferencia significativa entre el nivel de conocimiento en etiopatogenia y el año de estudio, con un valor de p = 0,005. El nivel de conocimiento sobre etiopatogenia fue regular en tercer año, bajo en cuarto año y regular en quinto año.

## Fuente de financiamiento:

Autofinanciado
